# Facebook increases political knowledge, reduces well-being and informational treatments do little to help

**DOI:** 10.1098/rsos.240280

**Published:** 2024-10-09

**Authors:** Kevin Arceneaux, Martial Foucault, Kalli Giannelos, Jonathan Ladd, Can Zengin

**Affiliations:** ^1^ Center for Political Research at Sciences Po (CEVIPOF), Paris 75007, France; ^2^ Paris-Dauphine University, Paris 75016, France; ^3^ Department of Government and MCourt School of Public Policy, Georgetown University, Washington, DC 20007, USA

**Keywords:** election, Facebook, news knowledge, subjective well-being, polarization

## Abstract

Nearly three billion people actively use Facebook, making it the largest social media platform in the world. Previous research shows that the social media platform reduces users’ happiness, while increasing political knowledge. It also may increase partisan polarization. Working to build a scientific consensus, we test whether the potential negative effects of Facebook use can be overcome with the help of minimalist informational interventions that a parallel line of research has shown to be effective at inducing people to be more accurate and civil. We conducted a pre-registered well-powered Facebook deactivation experiment during the 2022 French presidential election. In line with previous research, we find that deactivating Facebook increases subjective well-being and reduces political knowledge. However, deactivating Facebook had no overall effect on the level of political or social polarization during the election. Moreover, we find little evidence that minimalist informational interventions in a field setting helped individuals who deactivated Facebook to become better informed.

## 1. Introduction

In the space of the past two decades, optimism that social media platforms such as Facebook would provide a deliberative space for the free exchange of ideas and give a tool for political organizing to the less powerful and oppressed [1,2] has given away to deep pessimism about the deleterious social and political effects of social media [3,4]. Observational studies suggest that social media use is addictive and increases anxiety and depression [5,6], exposes people to false information [7], degrades users’ knowledge about politics [8] and deepens affective polarization [9,10]. Yet as is the case with all observational studies, these correlations could reflect omitted variables or, particularly in the case of social media where people curate their digital experience, selection bias.

Randomized field experiments, which use random assignment to isolate causal effects in real-world settings, address limitations of the internal validity of observational studies while maintaining a high degree of external validity. Fortunately, before we conducted our study, we had access to two innovative field experiments that gave monetary incentives to a random sample of study participants to deactivate their Facebook accounts, while the rest of the participants were allowed to continue using Facebook as they normally would. While these studies do not necessarily generalize to all social media platforms, Facebook remains by far the largest social media network in the world with nearly three billion active users [11]. The first study [12] was conducted during the 2018 midterm elections in the United States (US) and found that giving up Facebook for a month increased happiness, reduced partisan polarization, but also reduced levels of political engagement and knowledge. The second study [13] was conducted in Bosnia–Herzegovina in the summer of 2019 during the week around Remembrance Day (11 July), which commemorates the genocide of 8372 Bosnian Muslim men and boys in Srebrenica at the hands of the Bosnian Serb Army during the Bosnian War (1991−1995). It also found that those who gave up Facebook were happier and less informed but (unexpectedly) *more* polarized towards their ethnic outgroup. (Since we conducted our study, we learned of a similar Facebook deactivation study conducted during the 2020 US Presidential election that found smaller, statistically insignificant effects with respect to affective polarization and news knowledge [14].)

Taken together, these two field experiments [12,13] converge on the implication that, contrary to concerns about misinformation on social media platforms, people learn accurate information about politics from Facebook, but at the potential cost of their own subjective well-being. At the same time, these findings leave open to question whether Facebook polarizes. The results from the US study [12] corroborate survey-based experiments in the American context that consistently find that counter-attitudinal political posts on social media (and Facebook, in particular) polarize users [15–19]. By contrast, the Bosnia–Herzegovina study [13] measured social polarization (in terms of ethnic identities) as opposed to partisan polarization, and it took place during the commemoration of inter-ethnic violence. Perhaps, as the research team surmises, those in the control group in the Bosnia–Herzegovina study encountered on Facebook expressions of contrition by members of other ethnic groups, while those in the treatment group were more likely to only have conversations with co-ethnics, sharing memories about the atrocities committed by other ethnic groups towards their own. Or perhaps, posts about partisan disagreements are done in a more provocative and outrageous way than posts about ethnic differences. Another possibility is that cultural, political or social differences between the US and Bosnia–Herzegovina explain the different results. Only additional research can provide insight into why these first two studies produced apparently divergent results.

Moreover, these studies simulate what would happen in a world where Facebook continues to exist when people quit Facebook but retain all of their ‘bad’ digital habits. Instead of mindfully seeking out news, for instance, many participants in the treatment group of the US study consumed more entertainment [12,20]. This approach is akin to paying people to consume fewer calories on a diet regimen without instructing them how eat in a healthy manner. It turns out that the digital analogue to public health announcements to eat more fruit and vegetables may help people engage with social media in a more healthy way. A parallel stream of research, largely conducted in more controlled laboratory or survey settings, shows that providing people with advice on how to better use digital media can motivate them to reach accurate conclusions as opposed to ones that reinforce their favoured positions or groups. These informational treatments, in turn, reduce levels of misinformation and polarization [21–23]. A striking feature about this stream of research is that these informational treatments are minimalist. Participants in these studies are merely requested to consider the accuracy of information or to observe norms regarding civility. If these types of treatments are effective, it suggests that people may actually want to use social media in ways that are more aligned with the utopian Internet envisioned by cyberoptimists, but they need to be given a nudge to do so.

Building on previous research, we conducted a field experiment during the 2022 French presidential election that gave study participants incentives to deactivate their Facebook accounts. Our study makes two main contributions. First, our research design built in a direct replication of the effect of Facebook deactivation on these specific outcomes (subjective well-being, news knowledge and polarization) reported by the two previously discussed Facebook deactivation experiments [12,13]. Direct replications are important because they do more than verify the empirical scope of previous findings, they also facilitate theoretical refinement by confirming or dis-confirming the scope of previous theoretical claims [24]. France is similar and different in many ways from Bosnia–Herzegovina and the US.^1^ With so few field experiments on this important topic (the effect of social media use), additional studies that vary some of the country characteristics are helpful for building a consensus in the literature. If we replicate the key findings of these studies, it offers further evidence that these effects of Facebook are robust across contexts. In addition, we include measures of both political and social affective polarizations, allowing us to shed light on the reasons for the discrepancies in previous studies with respect to the effects of Facebook on polarization.

Second, our experimental design extends previous research by integrating Facebook deactivation with informational treatments. A random subset of the participants that we incentivized to deactivate their Facebook accounts were exposed to minimalist informational messages aimed at encouraging balanced news search, inducing accuracy motivations, encouraging civility and increasing digital literacy. To revisit our diet analogy, our design allows us to estimate the effects of the diet regimen alone (direct replication of Facebook deactivation studies component) as well as the effects of the diet regime coupled with tips for healthy eating (informational treatments component).

Based on the reality that the participants in the deactivation group could continue to consume information on the Internet and other social media platforms, the four messages in the informational treatment group were crafted to mitigate potential negative effects of social media use and promote healthier digital habits (outside of Facebook). These messages were informed by efforts to foster a healthier digital public sphere that highlight the need for common ethical standards and interdisciplinary approaches to mitigate these negative effects [25] as well as ‘nudges’ to combat misinformation that previous research has shown to be effective [22]. These concise informational treatments aimed to help participants balance and benefit their media consumption by offering advice regarding three actions participants could take. The messages focused on: (i) addiction, highlighting the addictive nature of social media; (ii) privacy, emphasizing the public nature of online behaviour; (iii) misinformation, encouraging diverse and accurate information-seeking; and (iv) civility, fostering a tolerant and civil online environment (see the electronic supplementary material, appendix S2).

Admittedly, we took a heterogeneous approach because we had no firm ex-ante expectations regarding which message would be most effective. The rationale behind our general approach was that it was better to try out different informational treatments in the hope that together they would be effective, either cumulatively or because different messages resonated with different individuals. Why did we choose these particular informational treatments? The design of these messages was informed by the widely recognized adverse effects associated with social media use, with the goal of offering a healthy alternative to those who had deactivated Facebook and were now left to create (potentially, at least) new digital habits. The addiction message noted that social media can be addictive, leading to excessive use that negatively impacts mental health and well-being, and we encouraged participants to suppress social media applications on their phone, spend time with friends and family and seek out news and information offline. Our aim here was to boost subjective well-being as well as encourage people to seek out information outside of social media. We crafted the privacy message to remind participants about the public nature of online interactions, where users often overlook the visibility and potential consequences of their actions, and we encouraged participants to educate themselves about ways to ensure their privacy. The goal of this message was to make participants more mindful about the social nature of digital interactions, increasing subjective well-being and reducing affective polarization. Given the prevalence of misinformation in political discourse as well as research on digital literacy, we crafted this message in light of recent research [22]: participants were made aware that misinformation commonly circulates online and offered three ways to combat it (e.g. cross-check information). Our goal was to both motivate individuals to become more informed (counteracting the negative effects of deactivation on news knowledge) as well as reduce the influence of misinformation that participants may have encountered as they ventured onto other (non-Facebook) social media platforms (e.g. TikTok). Finally, we designed the civility message to counteract the potential ways in which lack of civility in online discourse can create hostile and intolerant environments. Our goal here was to encourage participants to approach social media messages with a cooler and more open mind, reducing affective polarization in the process.

We pre-registered hypotheses with several different research projects in mind, organized by different types of effects (https://osf.io/xt5zg/). We separated our hypotheses into different categories to obviate concerns about selective reporting and false positives. For this particular project, we pre-registered the following hypotheses that replicate and extend previous Facebook deactivation studies:

**hypothesis 1a (H1a)**: participants who deactivated their Facebook account will report higher levels of subjective well-being relative to participants in the control group;

**hypothesis 1b (H1b)**: participants who deactivated their Facebook account *and* received informational messages will report higher levels of subjective well-being relative to participants in the control group and those in the ‘deactivation only’ treatment condition;

**hypothesis 2a (H2a)**: participants who deactivated their Facebook account will report lower levels of news knowledge relative to participants in the control group;

**hypothesis 2b (H2b)**: participants who deactivated their Facebook account *and* received informational messages will report higher levels of news knowledge relative to participants in the ‘deactivation only’ treatment condition;

**hypothesis 3a (H3a)**: participants who deactivated their Facebook account will report lower levels of affective polarization relative to participants in the control group; and

**hypothesis 3b (H3b)**: participants who deactivated their Facebook account *and* received informational messages will report lower levels of affective polarization relative to participants in the ‘deactivation only’ treatment condition.

Because these hypotheses replicate and build on previous research, and our goal is to speak to these specific outcomes as opposed to the total effects of Facebook use, we do not believe that it is necessary to correct for multiple comparisons. Nonetheless, we provide all the relevant information in our pre-registration document, allowing readers to correct for multiple comparisons if they wish.

## 2. Research design

**Figure 1.**
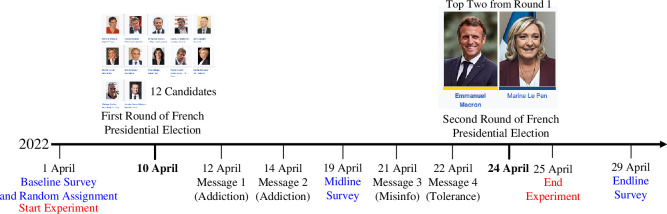
Timeline of Facebook deactivation study during the 2022 French presidential election.

We engaged IPSOS, a well-respected international survey research firm, to recruit French citizens who were eligible to vote and who reported that they regularly used Facebook. This group of IPSOS panelists were eligible to participate in the study if they said on the baseline survey (1 April 2022) they were willing to deactivate their Facebook account for several weeks in exchange for 80€. Among those who said they would be willing to do so, we then asked if they would be willing to be entered into a lottery where they had a 50% chance of being in the deactivation study that involved completing two additional surveys (for which they would receive 80€) or in a study where they would only take the two additional surveys in return for the regular incentive that the survey firm usually pays them for participating in surveys. Figure 1 illustrates the stages of the experiment.^2^ Of the 2246 respondents who gave their informed consent to participate in such a lottery, we randomly assigned 1117 to be in the deactivation treatment arm (547 randomly assigned to the ‘deactivation only’ treatment and 570 to the deactivation + information treatment). We designed the experiment to be powered to detect small treatment effects (d=0.2 at a power level of 0.95), and we ended up recruiting more participants than pre-registered, leaving us with an achieved power of 0.97 and the ability to detect very small treatment effects (d=0.18 at a power level of 0.95 and d=0.14 at a power level of 0.80). After participants completed the baseline survey, they were informed whether they had been randomly assigned to the deactivation treatment or the control group.^3^ Those assigned to the treatment arm were instructed how to deactivate their Facebook account, were reassured that deactivation would not delete their data, that they would continue to have access to Facebook Messenger throughout the study and that they could re-activate their account the day after the second-round election if they wanted.

France elects its president through a two-round system. The first-round election was held on 10 April 2022, featuring 12 candidates. Since none of the candidates received a majority of the votes, the top two vote recipients—incumbent President Emmanuel Macron (centrist) and Marine Le Pen (far right)—competed in the second-round election held on 24 April. Participants assigned to the deactivation + information treatment group received four messages between the two rounds of the election in a fixed order. Participants were surveyed two additional times after the baseline survey: the midline survey took place on 19 April (*n* = 1955, 13% attrition; we find no evidence of differential attrition across the experimental conditions; electronic supplementary material, appendix S4.3), between the first- and second-round elections, and the endline survey took place on 29 April (n=2246, no attrition), 5 days after the second-round election.^4^ See the electronic supplementary material, appendix S1 for descriptive statistics, S2 for the wording of the informational treatments, and S3 for the wording of outcome measures (in English and French).

In order to increase compliance with the experimental protocol, the research firm tasked employees with pinging the Facebook accounts of those assigned to the treatment arm to ensure that the accounts were deactivated. If an account had been reactivated, the research firm sent a message to the non-complying participant reminding them that they had agreed to deactivate their account for 80€, and requesting that they comply with the protocol. Unfortunately, we are unable to report exactly how many participants in the treatment arm failed to comply and needed to be sent a reminder message because the firm did not record this information. However, we did measure self-reported Facebook use on the midline and endline survey on a 5-point scale. The treatments reduced reported Facebook use considerably, but not entirely, in the midline wave (treatment – control = −1.1 for both treatment arms, p<0.01) and endline waves (deactivation only – control = 0.78; deactivation + information – control = 0.73, p<0.01). On the midline survey in which everyone in the treatment arm should have deactivated Facebook, 37.7% of the deactivation only group and 35% of the deactivation + information group reported not checking their Facebook feed at all as required by the experimental protocol, compared with just 0.01% of the control group, and over 70% in both treatment groups reported checking Facebook no more than once a day^5^ (see the electronic supplementary material, appendix S4.1, and table S5). Because these are self-reported responses and IPSOS-verified deactivation, it is possible that many participants misinterpreted the question to be about their normal Facebook behaviour. Yet, even if this were the true level of compliance, all experiments contend with noncompliance and it does not vitiate the internal validity of the design [26]. We calculate intent-to-treat effects that compare the behaviour of participants in the treatment groups and control group, irrespective of their compliance behaviour. We also note that we find no evidence of differential non-compliance between the two treatment groups (see the electronic supplementary material, appendix S4.2–S4.3). As a result, we estimate the unbiased effect of encouraging people to deactivate Facebook.

## 3. Results

For each outcome variable, we pre-registered the following ordinary least squares regression model for testing our hypotheses:


(3.1) $$y_{i}=\beta_{0}+\beta_{1}{DO}_{i}+\beta_{2}{DI}_{i}+\beta_{3}y_{iw=0}+e_{i},$$

where $y_{i}$ = the outcome variable for each participant i, ${DO}_{i}$ = an indicator variable 0, 1 for those assigned to the deactivation only treatment condition, ${DI}_{i}$ = an indicator variable 0, 1 for those assigned to the deactivation + information treatment condition, $y_{iw=0}$ = the outcome variable for each participant recorded in the baseline wave (if available) and $e_{i}$ = error term. For ease of interpretation, we report coefficient plots in the main text, but the regression models for all the analyses that follow can be found in the electronic supplementary material, appendix S5.

### 3.1. Subjective well-being

**Figure 2.**
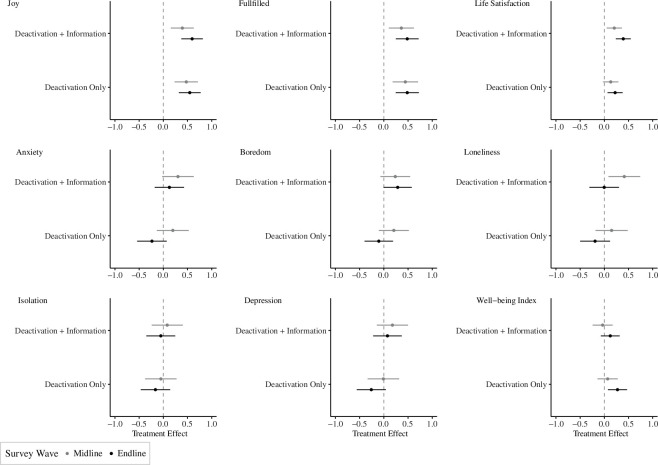
The effects of Facebook deactivation on subjective well-being.

Following previous Facebook deactivation studies [12,13], we measured subjective well-being by asking people to self-report mood over the past two weeks on an 11-point scale (ranging from 0 to 10) on seven discrete emotions (joy, fulfilment, anxiety, boredom, loneliness, depression and isolation) in addition to a general question about life satisfaction. We then averaged participants’ responses across all eight of these measures to create a well-being index. Figure 2 shows that, fully in line with previous research and H1a, by the end of the study, those in the deactivation only group rated their overall well-being, measured by the well-being index, more positively than those in the control group (d=0.05,p=0.007,Q=0.016; electronic supplementary material, appendix S5.1 and table S13). The results seem to be especially driven by people reporting that they felt more positive emotions (joy and fulfilled) as well as higher life satisfaction in both the midline and endline surveys across both treatment arms. For the deactivation only treatment, we do not observe a reduction in negative emotions in the midline. To the extent that Facebook deactivation reduced negative emotions, this is only evident by the end of the study. By contrast, we do not find consistent evidence for H1b. While the deactivation + information group also reported higher levels of positive emotions by the end of the study relative to the control group, it is not statistically significant (p=0.213,Q=0.396; electronic supplementary material, appendix S5.1, and tables S8–S11), and we do not observe that they report higher levels of well-being than those in the deactivation only group. If anything, by the endline survey, those in the deactivation + information reported feeling higher levels of anxiety and boredom than those in the deactivation only group (electronic supplementary material, appendix S5.1 and table S10).

### 3.2. News knowledge and political engagement

**Figure 3.**
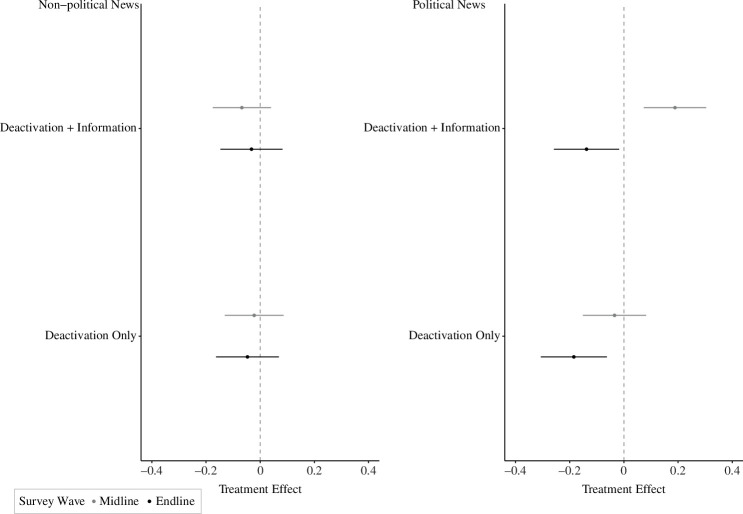
The effects of Facebook deactivation on news knowledge.

We measured news knowledge by asking participants on the midline and endline surveys to read 12 headlines and tell us whether each was true or false. Of the 12 headlines, six described events that had actually been reported in the news in the past week, while the other six were written in the style of a fake news headline: plausible, but false. In addition, for both the true and the false headlines, half were about politics and the other half were about non-political current events (e.g. entertainment and sports). This approach mirrors previous research [12,13], except that we measure both participants’ political and non-political news knowledge (see the electronic supplementary material, appendix S3.3). Figure 3 also corroborates previous Facebook deactivation studies and H2a: participants randomly assigned to the deactivation only treatment group were less knowledgeable about current political events on the endline survey (d=–0.07,p=0.003,Q=0.016; electronic supplementary material, appendix S5.2 and table S13). Interestingly, Facebook deactivation assignment had no effect on people’s knowledge about sports and entertainment events (non-political news, Bayes factors (BF) range from 0.06 to 0.11). Evidence for H2b is mixed. Consistent with our pre-registered hypothesis, participants assigned to the deactivation + information treatment knew more about politics on the midline survey relative to the deactivation only treatment and the control group (d=0.08,p<0.05; electronic supplementary material, appendix S5.2 and table S12). Yet, this effect completely dissipates by the endline survey where those in the deactivation + information group also knew less about political news than the control group, similar to the deactivation only group (p=0.025,Q=0.143). When we dig deeper, we find that the negative effects of the deactivation only treatment on news knowledge in the endline survey were largely driven by participants’ diminished ability to identify true political news. Facebook deactivation had essentially no effect on people’s belief in false (but plausible) news (${BF}_{deactivation}=0.06$, ${BF}_{information}=0.13$).

A possible explanation for the negative effects of Facebook deactivation on political news knowledge is that those in the deactivation only treatment group were less likely to consume political news. On both the midline and endline surveys, we asked participants how many minutes they spent ‘reading, watching or listening to news about politics’ and consistent with previous Facebook deactivation studies [12,13] participants in both the deactivation only and deactivation + information groups reported spending less time following the news in the previous week (p<0.05; see the electronic supplementary material, appendix S5.3 and tables S14 and S15). The fact that on the midline survey participants in the deactivation + information treatment condition were also less likely to follow the news in the previous week suggests that the informational treatments did not nudge people who deactivated Facebook to seek it out through other means (other social media, offline news, etc.). Although it is possible that the informational treatments boosted participants’ knowledge about politics on the midline survey through other means (e.g. discussion with friends), there is always the possibility that the observed positive effect is a ‘lucky’ draw from the null distribution.

### 3.3. Affective polarization

We asked participants on the midline and endline surveys to rate how they felt about members of different groups on an 11-point scale ranging from 0 (‘you don’t like them at all’) to 10 (‘you have a very warm feeling toward members of this group’). Similar to Allcott *et al*. [12], we measured partisan and ideological affective polarization by taking the difference between the rating of a participant’s ingroup (their preferred party or ideological group) and outgroup (their rating of their least preferred party or opposing ideological group). Similar to Asimovic *et al.* [13], we measured social affective polarization by taking the difference between participants’ feelings toward citizens without an immigrant background as well as their feelings toward citizens with a Maghreb and African immigration background, which represents an important social (cultural and religious) cleavage in France. Inconsistent with both H3a and H3b, we find that Facebook deactivation had essentially no effect on partisan polarization, ideological polarization or social polarization. Not only are all of the effects that we observed statistically insignificant (p>0.05), the effect size in almost every case is near zero (see the electronic supplementary material, appendix S5.4 and tables S16 and S17; BF range from 0.06 to 0.14).

### 3.4. Pre-registered heterogeneous effects

Our pre-analysis plan pre-registered four conditional average treatment effects. With respect to news knowledge, because education and age are positively correlated with news exposure [27], we hypothesized that Facebook deactivation would have a larger negative effect among college-educated respondents relative to non-college-educated respondents and that it would have a larger negative effect among older respondents relative to younger ones. With respect to partisan affective polarization, we hypothesized that because college-educated individuals tend to have higher levels of partisan affective polarization [28], Facebook deactivation would lower partisan affective polarization among this subgroup relative to non-college-educated individuals. Finally, with respect to social affective polarization, we hypothesized that Facebook deactivation would have a larger negative effect on individuals without a college education relative to those with a college education because education is negatively correlated with prejudice and ethnocentrism [29]. The results for these hypotheses are located in the electronic supplementary material, appendix S6 and tables S18–S21. We do not find robust evidence for any of these hypothesized conditional average treatment effects.

## 4. Discussion

**Figure 4.**
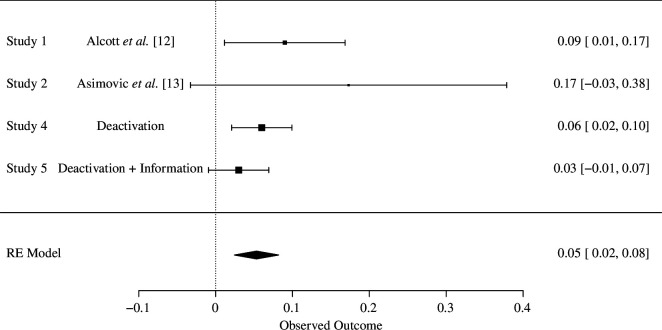
Forest plot for the effects of Facebook deactivation on subjective well-being. RE - Random Effects (RE) model.

**Figure 5.**
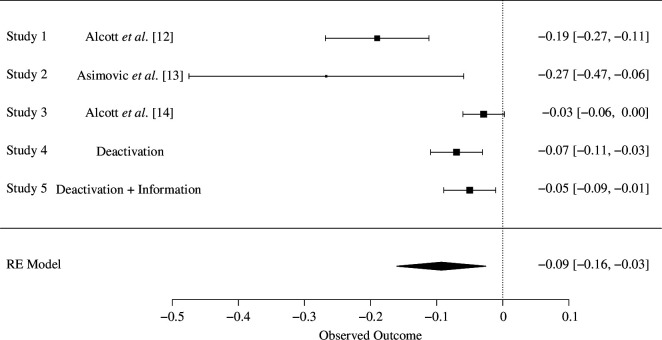
Forest plot for the effects of Facebook deactivation on news knowledge.

**Figure 6.**
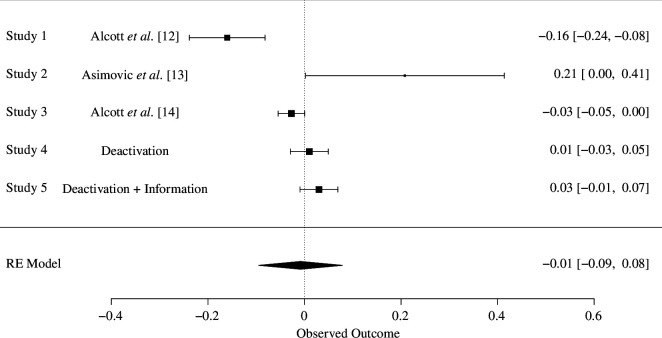
Forest plot for the effects of Facebook deactivation on polarization.

We conducted a Facebook deactivation study during the 2022 French presidential election. Our goal in this aspect of the project was to replicate and extend previous research that concentrated on the effects of Facebook deactivation on subjective well-being, news knowledge and polarization. Accordingly, it would be useful to consider our results in the context of previous findings, and so we display forest plots of the standardized effect sizes for each outcome in figures 4–6. The standardized effects sizes come from the main analyses reported in previously published research and from the results observed on our endline survey.

In summary, our results corroborate previous research with respect to well-being and news knowledge. As figure 4 shows, participants randomly assigned to deactivate their Facebook account expressed slightly higher levels of subjective well-being (as measured by the index) than those assigned to the control group. Across these studies, including ours, a random effects model estimates that deactivating Facebook increases subjective well-being by 0.05 s.d. and that this effect is distinguishable from no effect. In terms of the substantive effect of Facebook deactivation on subjective well-being, a 0.05 s.d. effect is consistent with the ‘smallest effect size of interest’, meaning that individuals would recognize that they feel slightly better than before [30].

Figure 5 shows that across previous studies and this one, individuals assigned to deactivate their Facebook account were less informed about politics than those randomly assigned to the control group who continued to have access to Facebook. A random effects model estimates that deactivating Facebook decreases knowledge about politics by 0.09 s.d., which is a small but substantively meaningful effect [31]. While we cannot be sure about the mechanism that explains why people who deactivate Facebook know less about politics, these findings are consistent with the notion that social media, and Facebook in particular, provide political information to people who do not actively seek out the news. Before the rise of social media, the expansion of entertainment choices in the media environment caused by the rise of cable television and the Internet allowed people who were not very interested in news to avoid it, or at least not actively seek it out, which increased the gap in political knowledge between news-seekers and entertainment-seekers [32] and blunted the reach and effects of the news media [33]. The social nature of social media, though, means that people are often exposed to information, including information about politics, that they may not seek out [19]. If their friends are posting about a political event, such as an election, they will learn about it even if they do not regularly read the newspaper or watch news on television, for instance. In this way, social media allows news to find people [15,34]. Our findings coupled with those from previous deactivation studies are consistent with the argument that, among the group of people who depend on news to find them, giving up Facebook diminishes their knowledge about politics.

In contrast to the research on the topic that inspired our replication [12,13], we find little evidence that Facebook deactivation has an overall effect on affective polarization. These null results are consistent with a recently published Facebook deactivation study that was conducted during the 2020 US presidential elections [14]. A random effects model estimates the pooled effect of Facebook deactivation on polarization as centred on zero (figure 6).

Turning to the effects of our informational treatments, we find modest evidence that our informational treatments helped participants in the deactivation treatment arm to be more accurate when evaluating political news headlines. We only found positive results in the midline survey, and these effects dissipated by the endline survey. To the extent that our informational treatments were effective at increasing news knowledge, they did so by boosting participants’ knowledge about true news, as opposed to enhancing their ability to identify plausible but false news. Beyond news knowledge, and contrary to our pre-registered expectations, we find little evidence that informational treatments aimed at encouraging people to be civil and recognize the addictive nature of social media caused participants to be happier than participants who deactivated their Facebook account but did not receive informational treatments. Indeed, the forest plots show that across all of our outcomes, the deactivation + information treatment effects did not differ statistically from the deactivation treatment effects, on average.

Overall, our results suggest that claims about the effectiveness of these types of information treatments should be treated with caution. Our pre-registered expectations about the effects of these informational treatments produced mostly null results. While it is certainly possible that ‘better crafted’ informational treatments would have ‘worked’ for everyone, we note that our informational treatments were no different in terms of quality or content from those employed in previous survey experiments. We also note that in studies where informational treatments increase news accuracy or digital literacy, the researchers measured outcomes right after delivering the information. Thus, our findings could be consistent with the explanation that these informational treatments decay quickly and are unlikely to motivate sustained behavioural change [35]. Nonetheless, we cannot rule out that either our potpourri approach or the specific messages that we crafted were simply ineffective at influencing the behaviours that we had hoped to alter (e.g. news-seeking behaviour), and that another approach—such as daily messages—would work better.

We believe that reproducing previous research findings regarding subjective well-being and news knowledge is important for two reasons. First, it bolsters confidence in the generalizability across different societies of the claim that social media use makes people less happy but also more informed about politics. These findings appear to be robust across different cultural contexts as well as time. Second, it bolsters our confidence in the integrity of the deactivation + information treatment. To the extent we find null effects when we go beyond previous work, we are less concerned that our failure to administer the treatments properly is to blame. Replication also provides clarity on magnitude of the effects of interventions. In this respect, we find smaller effect sizes on news knowledge and well-being than in the previous studies in the US and Bosnia–Herzegovina. In terms of substantive importance, then, our results suggest that if people were to give up Facebook without any incentives, it would potentially make the world a slightly happier and slightly less politically informed place.

Future research should focus on better understanding the mechanisms that shape the influence of social media (or its absence) as well as the degree to which informational treatments and digital literacy nudges can be strengthened in real-world settings. A key limitation of our experiment, and of all Facebook deactivation designs, is that it is a blunt instrument for studying the effects of social media use. In contexts where social media are ever-present, as is the case in the contexts where Facebook deactivation has been studied to date, deactivation experiments offer insights into what happens when people decide, based on external incentives, to hit the pause button. It is difficult to know what the effects of deactivation would be under other conditions, especially if many people intend to return to Facebook after the end of the study. For these individuals, the deactivation protocol simulates a temporary hiatus as opposed to an intended lasting change in behaviour. As such, perhaps these individuals felt less need to find new habits. Moreover, these designs can only make inferences to the population of Facebook users who are willing to deactivate their accounts for the amount of money we offered as an incentive. It is impossible to know if deactivation would have different effects on those who did not wish to participate. Another key limitation to these studies is that they do not enable us to fully explore the social nature of social media effects. Future research could address this issue by encouraging groups of friends to deactivate their social media accounts, for instance. Finally, our research, like previous research, focuses on an environment in the northwestern hemisphere, and we should be cautious when it comes to making inferences about the influence of social media in other regions of the world.

## Data Availability

The data and code necessary for replicating all statistical analyses and code reported in the main text and electronic supplementary material can be found on the OSF pre-registration page for the project and the following [36]. The replication code is contained in R markdown files, requiring R Studio to run. Supplementary material is available online [37].

## Footnotes

^1^
Like the US, France is a western democracy with a presidential system, and similar to Bosnia–Herzegovina, the major political cleavage in France is ethno-religious (northern African immigrants and their descendants who tend to identify as Muslims). Yet, there are important differences as well. France, unlike the US, has a multi-party system and political polarization that does not reflect two opposing, equally sized party coalitions and unlike Bosnia–Herzegovina where Muslims and Christians each make up roughly 50% of the population, Christians and non-religious people in France far outnumber the adherents of Islam. In this way, France offers a less extreme case with respect to social and political polarizations while having important political cleavages nonetheless.

^2^
Social science research in France is exempt from seeking ethical approval. We followed closely the research design of previous studies that did receive Institutional Review Board approval in the United States, and participants had to provide their informed consent.

^3^
The informational treatments are not fully crossed in the experimental design for two reasons. First, we were most interested in testing the hypothesis that the informational treatments would help people adjust their media habits in a healthy way when they deactivate Facebook. Second, our budget did now allow increasing the sample size; thus, adding a fourth experimental condition would have decreased the statistical power of the study.

^4^
Because IPSOS only paid participants if they participated in the endline survey, we were able to achieve a 100% participation rate on that survey.

^5^
With respect to the informational treatments, we asked participants randomly assigned to the deactivation + information group if they ‘received informative messages by email indicating the negative effects of social media and online platforms?’ Of the 570 participants, 74% reported that they had, 72.6% reported that they read them and 68.6% reported that they followed their suggested guidelines.
